# Synthesis of Vertical Carbon Nanotube Interconnect Structures Using CMOS-Compatible Catalysts

**DOI:** 10.3390/nano10101918

**Published:** 2020-09-25

**Authors:** Zichao Ma, Shaolin Zhou, Changjian Zhou, Ying Xiao, Suwen Li, Mansun Chan

**Affiliations:** 1 Dept. Electronic & Computer Engineering, The Hong Kong University of Science and Technology, Hong Kong, China; zmaaa@connect.ust.hk (Z.M.); zhoucj86@gmail.com (C.Z.); yxiaoal@connect.ust.hk (Y.X.); suwen.li@connect.ust.hk (S.L.); mchan@ust.hk (M.C.); 2 School of Microelectronics, South China University of Technology, Guangzhou 510640, China

**Keywords:** carbon nanotube, CMOS-compatible, catalyst design, interconnect

## Abstract

Synthesis of the vertically aligned carbon nanotubes (CNTs) using complementary metal-oxide-semiconductor (CMOS)-compatible methods is essential to integrate the CNT contact and interconnect to nanoscale devices and ultra-dense integrated nanoelectronics. However, the synthesis of high-density CNT array at low-temperature remains a challenging task. The advances in the low-temperature synthesis of high-density vertical CNT structures using CMOS-compatible methods are reviewed. Primarily, recent works on theoretical simulations and experimental characterizations of CNT growth emphasized the critical roles of catalyst design in reducing synthesis temperature and increasing CNT density. In particular, the approach of using multilayer catalyst film to generate the alloyed catalyst nanoparticle was found competent to improve the active catalyst nanoparticle formation and reduce the CNT growth temperature. With the multilayer catalyst, CNT arrays were directly grown on metals, oxides, and 2D materials. Moreover, the relations among the catalyst film thickness, CNT diameter, and wall number were surveyed, which provided potential strategies to control the tube density and the wall density of synthesized CNT array.

## 1. Introduction

**Figure 1:**
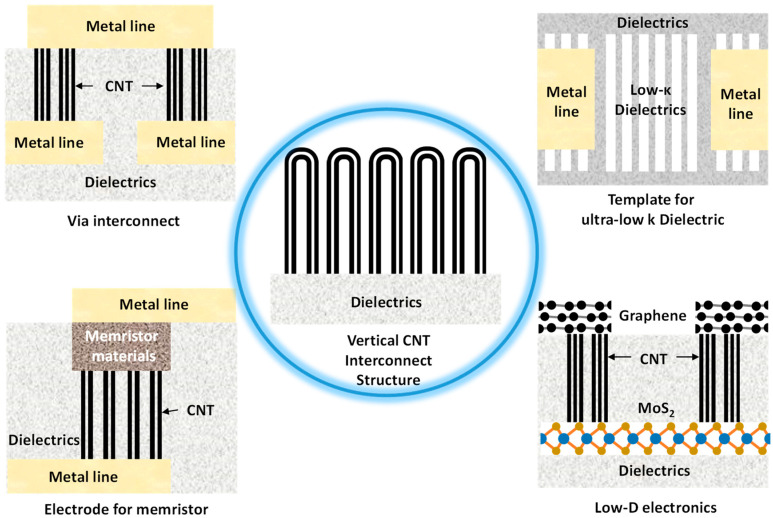
Applications of the vertically aligned carbon nanotube (CNT) structure.

Vertically aligned carbon nanotubes (CNTs), which show high conductivity, high thermal stability, and high-mechanical strength [1,2,3], have been intensively studied and employed as the electrodes and interconnects for advanced interconnect applications [4,5,6,7,8]. As a single CNT can provide a high current density close to 10^9^ A/cm^2^ [5], the CNT array with a wall density more than 10^13^ cm^−2^ has been considered as the ideal material for the via interconnect to overcome the electromigration challenges in current complementary metal-oxide-semiconductor (CMOS) technology [9,10,11,12]. As shown in Figure 1, besides the via interconnect applications, the vertically aligned CNT interconnect structure was proposed as a vital component in all-carbon interconnects and low-dimensional (low-D) electronics [13]. The highly ordered CNT array also serves the ideal template to fabricate low-κ dielectrics with quasi-periodic and porous features. By using the process of CNT array templating [14], dielectric layers with a recorded low value of 1.75 were achieved [15]. By concentrating the current and electric field in the wall of the CNT with a nanometer-level diameter, the CNT electrode showed the potential to reduce the operation voltage, improve the device stability and enable ultra-dense integration of memristors [16,17,18].

Applying the CNT-template interconnect structure on a chip requires the CNT array grown on metallic and insulating substrates, making the direct synthesis of the vertically aligned CNT array using a CMOS compatible procedure becomes essential. As a promising candidate for interconnect applications, CNT array grown with high tube density, high wall density, and high lattice integrity is desirable to fabricate the low-resistivity conduction lines [19,20]. Therefore, methods to control the density and the diameter of the synthesized CNT array are indispensable for the CNT-based interconnect technology. To minimize the effect of the thermal fluctuation on the active region and dielectric layers, the process to synthesize CNT interconnect needs to be conducted at temperatures below 450 °C [19,20]. 

In the CMOS-compatible regime, chemical-vapor-deposition (CVD) is a popular method to grow CNT using the metal catalysts that can be deposited and removed without contamination using standard CMOS processes. Commonly used CMOS-compatible catalysts include nickel (Ni), cobalt (Co), copper (Cu), aluminum (Al), and titanium (Ti). However, the popular iron (Fe) catalyst causes contamination to the CMOS fabrication process, which is not a choice to synthesis the CNT interconnect structures using CMOS applications. Recent works reported and revealed that CNT array can be reliably synthesized with high-density on metallic and insulating substrates, using the metal catalyst of nickel or cobalt [21,22,23,24]. Other works also explored approaches to reduce the CNT growth temperature while maintaining a high array density [25,26,27,28,29]. It turns out that the catalyst design has played a vital role in CNT synthesis. At the same time, the carbon gas-precursor, the substrate underneath, and the process condition are also essential factors that affect the CNT growth. Moreover, the integration of CNT interconnects to atomic-level thin 2D materials were also reported [30,31,32]. 

With the updated knowledge of state-of-art technology of CNT synthesis using CMOS-compatible methods, this paper is devised into three parts. The first part addressed the synthesis of high-density CNT array, which is essential to the CNT interconnect technology. The second part summarized the strategies to reduce the synthesis temperature, which have a similar idea of lowering the activation energy of CNT growth. Finally, a few reports on the synthesis of CNT on 2D materials are reviewed. 

## 2. Synthesis of High-Density CNT Array

In principle, the CNT array with high density is essential to reduce the resistance for the interconnect applications. For the interconnect technology that aims to exceed the performance of the existing Cu interconnect, Awano et al. [19] pointed out the required minimum wall density of 10^13^ cm^−2^. Moreover, the vertical CNT array synthesized with high-density has additional advantages in that the self-organized nanotubes bundles are grown to be perpendicular to the substrate [33]. Compared to the CNT array with a low density, the self-organized high-density CNT array demonstrates less deformation, buckling, and defects. 

Overall, the generation of catalyst nanoparticles with high density is the preliminary step for high-density CNTs synthesis. However, diffusion of the metal catalyst into the substrate is a common problem that hinders the formation of uniform catalyst nanoparticles [34,35], which results in sparse CNT growth. Many works that used Co or Ni catalyst films for CNT synthesis had reported similar problems [27,29,36]. As a result, forming a diffusion barrier to the catalyst film turns out to be a feasible solution. Elemental metals that are d-orbital vacancies-rich, such as Al, Mo, and Ti [27,29,32,36], were popular choices of diffusion barrier by adding an atomic-layer thin film in between the catalyst and the substrate. Sugime et al. [27] simply added an Al film with a thickness of 5 Å in between the catalyst film and the substrate and found out that the diffusion of Co catalyst was prevented. Herein, catalyst nanoparticles with an areal density of 6–8 × 10^11^ cm^−2^ were found to be effectively generated by thermal annealing. The critical mechanism that improved catalyst nanoparticle formation was interpreted as the alloy formation between the Al layer and the substrate.

Meanwhile, the Al layer is prone to form an alloy with the catalyst particles as well, and the thickness of the Al layer that determined the atomic ratio of Al in the formed catalyst nanoparticles significantly affects the CNT growth. Sugime et al. [27] found out that by using an Al film of 2.5 Å below the Co catalyst film, the growth rate of the CNT array became much smaller than that using a 5 Å Al film. On the contrary, with a 1 nm Al film grown underneath the Co catalyst, the CNT growth was substantially hindered, resulting in sparse density and distorted morphology. Similar effects of deposition of metal layers together with catalyst film were observed in [29,36]. 

**Figure 2:**
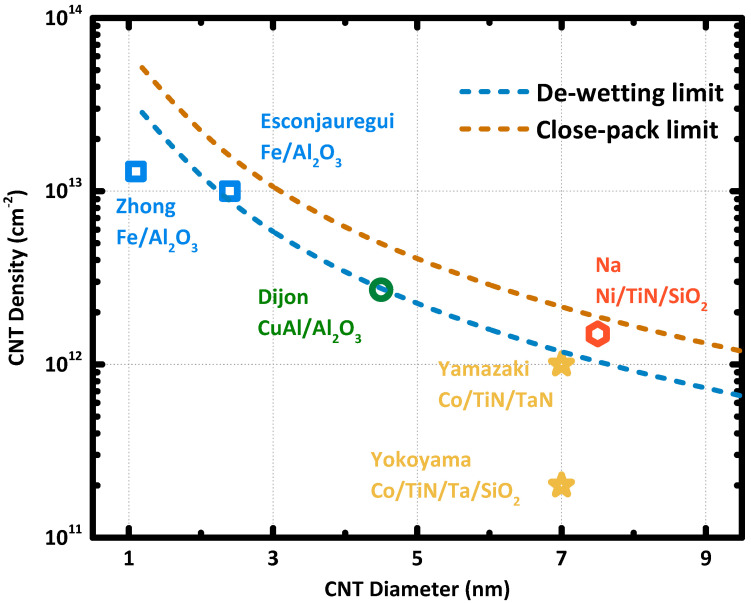
The close-pack limit and de-wetting limit are replotted using the equations in [26]. The experiment results of high-density CNT array and the average CNT diameter. Data points are obtained from [29,36,37,38,39]. The catalyst and substrate are marked as catalyst/substrate.

Zhong et al. [26] proposed a theoretical limit of CNT array density with an average nanotube diameter. As shown in Figure 2, the highest tube-density is achieved in the close-pack scheme, which requires the CNTs to be close contact with the adjacent tubes in an array. Achieving the close-packed CNT array is unrealistic since the de-wetting of catalyst nanoparticle generates innate spaces between the nanoparticles. Therefore, the proposed de-wetting limit [26], as shown in Figure 2, is more instructive to benchmark the density of CNT array according to their average tube diameter. However, as the de-wetting of the catalyst nanoparticle depends on the metal element, substrate, and chamber condition, the curve of the de-wetting limit can vary for different CNT growth processes. However, due to the complexity of catalyst nanoparticle de-wetting, it is very challenging to predict the de-wetting limit in theory for CNT growth using a specific metal catalyst on arbitrary substrates. Some works reported CNT density exceeding the proposed de-wetting limit [37,38,39]. Notably, in Figure 2, Na et al. [39] had synthesized CNT array with a nanotube density of 1.5 × 10^12^ cm^−2^ and an average tune diameter of 10 nm, which readily exceeded the de-wetting limit proposed in [26]. 

**Figure 3:**
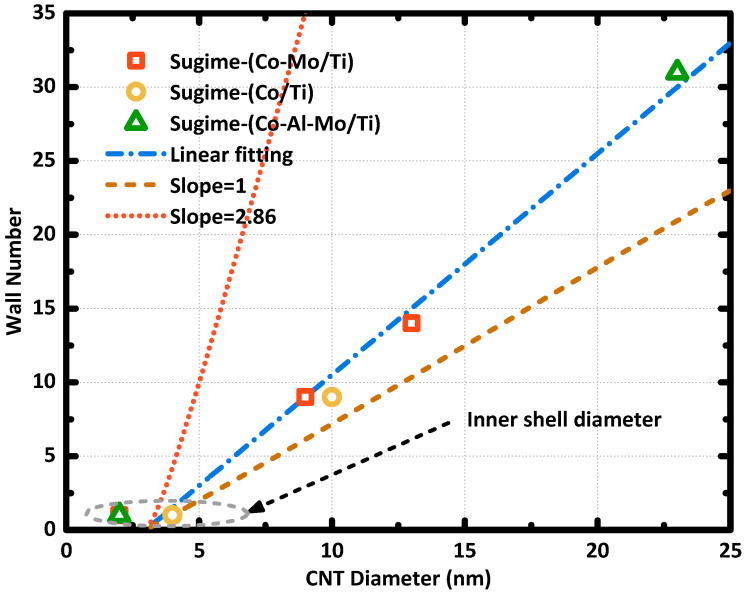
The relation between the average CNT diameter and the average wall number, data points are obtained from [27,29]. The catalyst and substrate are marked as catalyst/substrate.

However, it is also seen that the synthesis of CNT array with tube density exceed 10^13^ cm^−2^ is challenging, which is particularly true when using a CMOS-compatible catalyst. While on the other hand, most of the reported CNT with a tube diameter of several nanometers to a few tens of nanometers had a multiwall structure. The multiwall structure of the synthesized CNT provides a chance for the CNT array to satisfy the wall-density requirement of interconnect applications. The relationship between the CNT diameter and wall number were surveyed. Figure 3 summarizes the linear relations between tune diameters and wall numbers reported in previous literature [27,29]. The linear relation is highlighted by the blue fitting line, which shows a slope larger than 1, and it is intercepted to the *x*-axis due to the hollow inner shells that usually have diameters around 3 nm. The line with a slope of 2.86 revealed the relation between CNT wall number and tube diameter when the CNT walls were arranged with a graphite structure, where the carbon layer has a thickness of 3.5 Å. The gentler slope of the fitting curve suggested a sparse package of carbon walls in the multiwall CNTs. Even though the fitting of experimental data already indicated the possible synthesis of multiwall CNT array with wall density around 10^13^ cm^−2^, the effect of the metal catalyst and the substrate on determining the relation between the wall number of the CNT and tube diameter was not systematically investigated. 

**Figure 4:**
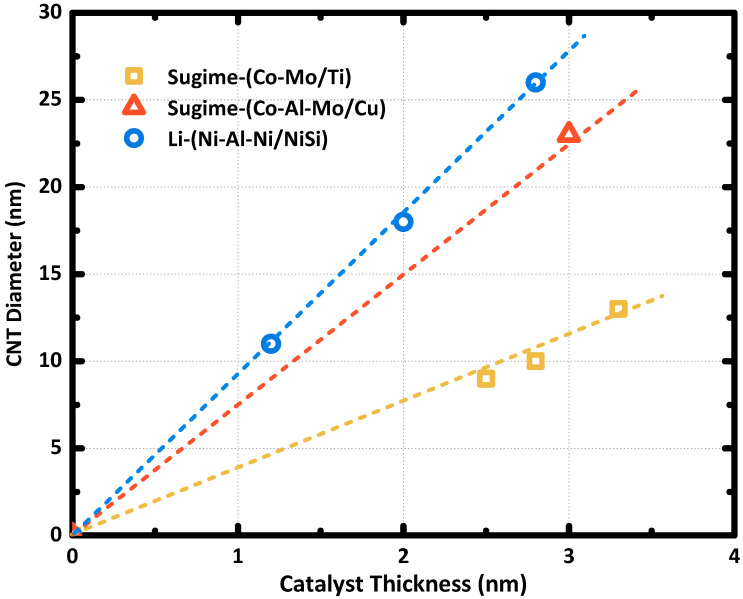
Catalyst film thickness dependent average CNT diameter. Data points are obtained from [27,29,42]. The catalyst and substrate are noted in catalyst/substrate.

The thickness of the catalyst film was found to determine the average nanotube diameter. Interestingly, characterizations showed that the CNT density follows a linear relationship with the thickness of catalyst film as well, and the slope of the linear relation can be modified by the CNT synthesis process [40,41]. As shown in Figure 4, the linear relation between the catalyst film thickness and the average CNT diameter was determined according to results reported in the literature [27,29,42]. The catalyst element, substrate support, and the process conditions clearly showed a significant effect on that relationship, which indicated the randomness of the catalyst nanoparticle formation, as each CNT is individually grown in one single catalyst nanoparticle. Systematic experiments are still necessary to uncover the relations among CNT density, catalyst nanoparticle density, catalyst film thickness, substrate material, and process condition. Bedewy et al. [43] characterized the de-wetting process of nanoparticle formation upon heating by the in-situ transmission electron microscope, which showed that the catalyst nanoparticles are formed within a few seconds. Therefore, the short-term process variation at the initial annealing step has an essential effect on the uniformity of the catalyst nanoparticle formation and, consequently, the morphology of the synthesized CNT array. 

The issue of partial activation of catalyst nanoparticle that leads to reduced CNT density was also widely observed. Na et al. [39] characterized the catalyst nanoparticles with an area density of 2.8 × 10^12^ cm^−2^, while the synthesized CNT array showed a density of 1.5 × 10^12^ cm^−2^, which indicated that only half of the generated catalyst nanoparticles were activated to facilitate the CNT growth. As generating a catalyst nanoparticle with a high area density is already a challenge, increasing the ratio of active catalyst nanoparticle is particularly important to synthesize CNT array with a density of 10^12^ to 10^13^ cm^−2^. To seek clues on improving the ratio of active catalyst nanoparticles, Aguiar-Hualde et al. [44] simulated the process of CNT growth with Ni catalyst nanoparticle by molecular dynamics method and interpreted the whole CNT growth process as a de-wetting process. They found that the charge polarity of catalyst nanoparticle had a substantial effect on the catalytic activity. Specifically, positively charged Ni nanoparticle was found prone to be deactivated by carbon network encapsulation [44], which was due to the low carbon solubility that allowed only a low carbon fraction in the nanoparticle. As a result, the Ni^+^ nanoparticle tended to adhere to the carbon network with a small contact angle and eventually be encapsulated by carbon networks. 

To avoid the catalyst deactivation, the alloyed Ni catalyst, in which the Ni atom was negatively charged, was also suggested [44]. By adding a few atomic layers of Mo to the Co catalyst film, Sugime et al. [29] reported that the ratio of active Co catalyst nanoparticles was increased to 80%. In contrast, the pure Co film only generated 35% active catalyst nanoparticles. On the other hand, adding a trace amount of water vapor to the CNT growth process was demonstrated to prolong the active lifetime of the catalyst, which improves the uniformity of the synthesized CNT array [45,46]. The mechanism of removing the carbon coating around the catalyst nanoparticles [47,48] is an enlightening strategy for increasing the ratio of active catalyst nanoparticle as well. Recently, Dee et al. [49] proposed to pretreat the catalyst nanoparticles with trace exposures of carbon during the initial stage of annealing of the catalyst film. By optimizing the duration of carbon exposure, they achieved 8 times higher CNT density relative to a reference growth process condition. Moreover, Wu et al. [50] and Zhou et al. [51] reported the catalyst nanoparticles deactivated in one cycle of CNT synthesis could be reactivated in another growth cycle. Through synthesizing CNTs in three growth cycles, the density CNT array was increased by four times [51]. 

The record-high density of CNT array that was synthesized in the CMOS-compatible process was reported by Na et al. [39] as 1.5 × 10^12^ cm^−2^. The CNT array was synthesized using Ni catalyst with a nominal film thickness of 0.6 nm on SiO_2_ substrate. To prevent the thin Ni film diffusion into the SiO_2_ substrate, a 5 nm TiN film was inserted as the barrier layer. Moreover, Na et al. pointed out the importance of using catalyst metal film with fine grain size to synthesize the high-density CNT array. 

## 3. Low-Temperature Synthesis of CNT Array

**Figure 5:**
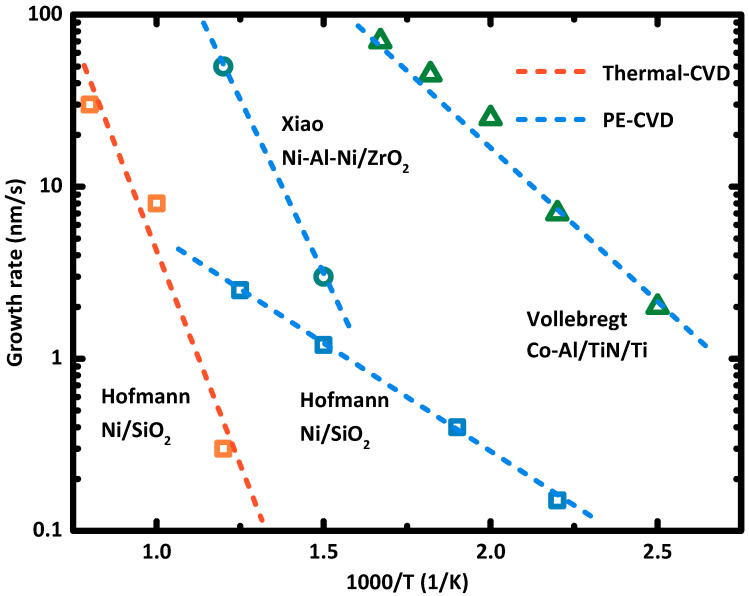
Activation energy of CNT growth determines the relation between CNT growth rate and growth temperature. The catalyst and the substrate are marked as catalyst/substrate. Data points are obtained from [14,48,53].

As shown in Figure 5, the growth rate of CNT to the process temperature was described by the Arrhenius plot. The growth rate of CNT decreases exponentially with 1/T, where the T is the absolute temperature. The activation energy of CNT growth determines the slope of the line. The slower growth rate often indicates the lower catalyst activity of the catalyst nanoparticle, which can lead to more deactivation of the catalyst nanoparticle and result in worse CNT growth. Therefore, high temperature is usually necessary to guarantee CNT growth with a fast growth rate of 10 to 100 nm per minute. To synthesis CNTs at low temperatures, reducing the activation energy of CNT growth is essential. Hofmann et al. [52] demonstrated the efficacy of activation energy reduction using the plasma-enhanced chemical vapor deposition method, which is now a popular method to grow CNT array at low temperatures. Moreover, they concluded that the limiting mechanism in plasma-enhanced CNT synthesis was the carbon diffusion on the surface of catalyst nanoparticle, which indicates that catalyst design is the key to grow CNT at low temperatures. 

Enhancing the low-temperature activity of the catalyst by using multilayer metal thin films to form the catalyst nanoparticle is a popular approach. Synthesis of vertically aligned CNT array with tube-density from 10^11^ to 10^12^ cm^−2^ at process temperatures of 400 to 450 °C was intensively reported [27,28]. While only a few works achieved the synthesis of CNT at 350 °C using CMOS-compatible methods. Vollebregt et al. [49] reported the synthesis of vertically aligned CNT array at 350 °C using the Co-Al alloy catalyst. At 350 °C, the CNT array synthesized with Co-Al catalyst showed a tube density of 5 × 10^10^ cm^−2^. Compared to the CNT synthesis using pure Co catalyst, the author found that CNT synthesized with Co-Al catalyst showed a faster growth rate and better vertical alignment. In the Arrhenius plot, the Co-Al catalyst showed lower activation energy (0.4 eV) than that of the pure Co catalyst (0.43 eV). 

Similarly, Li et al. [36] adopted the Ni/Al/Ni tri-layer architecture to form the Ni-Al alloy catalysts. The CNT array was synthesized at 350 °C with a tube density of 2 × 10^10^ cm^−2^ and a growth rate of 75 nm/s. In contrast, the CNT synthesized with pure Ni catalyst at 350 °C only showed a lower tube density of 5.9 × 10^9^ cm^−2^ and a slower growth rate of 33 nm/s. Xiao et al. [15] reported the synthesis of CNT array using plasma-enhanced chemical vapor deposition (PECVD) and the CMOS-compatible metal catalyst at a lower temperature of 340 °C. The vertical CNT array was synthesized on the ZrO_2_ substrate using the Ni/Al/Ni (1 nm/0.5 nm/1 nm) triple-layer catalyst design, which turned out to be the same as that in [28,36]. The tube-density of the CNT array that was synthesized at 340 °C was characterized to be around 10^12^ cm^−2^, and the activation energy for CNT growth using the Ni-Al-Ni catalyst on ZrO_2_ substrate was extracted to be 0.35 eV. 

Moreover, the substrate support has shown a significant effect on the catalytic activity of the Ni-Al-Ni catalyst [14]. Xiao et al. found that the Ni-Al-Ni failed to facilitate CNT synthesis on SiO_2_ substrate or Al_2_O_3_ substrate even at a higher temperature of 450 °C [14]. Moreover, Ahmed et al. [50] even reported that the thickness of the metal substrate determines the morphology, average tube diameter, and tube density of synthesized CNT array. The comparative experiments suggested that the substrate type exerts a significant effect on the CNT growth at low temperatures. 

Preserving the lattice quality of the CNTs when synthesized at low temperatures is also highly demanded for the interconnect application. The lattice quality of CNT can be implied by the intensities of the D-band signal and G-band signal in the Raman spectrum. The reported CNTs that synthesized below 450 °C show an I_D_/I_G_ of 0.8–1.2, the large value of I_D_/I_G_ indicates a high density of defects formed in the CNT. The high defect density in CNTs can be caused by the plasma particles in the synthesis process [14,28,53]. Therefore, a method to reduce the activation energy of CNT growth but not to generate energetic and destructive particles is highly desirable. Ahmed [54] proposed a photothermal CVD method to synthesize CNT with high crystallinity at low temperatures. In this method, an array of optical lamps was arranged inside the chamber to reduce the activation energy via photon energy. The CNT array with the areal density of 10^10^–10^11^ cm^−2^ was grown at 350 to 440 °C, with the D-band to G-band intensity ratio characterized by Raman spectroscopy to be as low as 0.13 [54]. 

## 4. Direct Growth of CNT on 2D Materials Substrates

Synthesis of CNTs on graphene is of particular interest for the all-carbon interconnect technology and all-carbon electronics [13,19]. By embedding the vertically aligned CNT into horizontal graphene and other 2D-material based electronic devices, the planar low-dimensional electronics can be integrated into a three-dimensional hierarchy. In this section, recent works on the synthesis and characterizations of vertical CNT array on 2D materials are summarized. 

**Figure 6:**
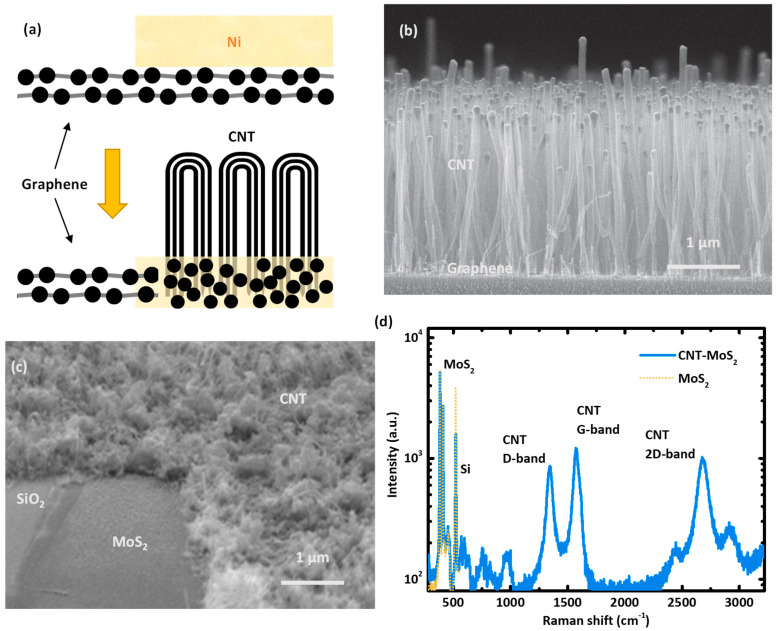
(**a**) An illustration of the Ni-C alloy formation in CNT–graphene structure synthesis. (**b**) An SEM image of the vertically aligned CNT array grown on the graphene/SiO_2_ substrate [31]. (**c**) An SEM of the vertically CNT array grown on MoS_2_/SiO_2_ substrate [32]. (**d**) The Raman spectra of CNT arrays grown on MoS_2_/SiO_2_ [32]. (Reproduced with permission from [31], Institute of Physics (Great Britain), 2016) (Reproduced with permission from [32], Institute of Electrical and Electronics Engineers, 2019).

Nickel has been the popular choice to synthesize CNT–graphene interconnects structure. As illustrated in Figure 6a, the high solubility to carbon and the low temperature required to form the carbide alloy makes Ni a unique catalyst to synthesize the CNT interconnect on graphene with low contact resistance [55,56]. Ryu et al. [30] reported the direct synthesis of vertically aligned CNT array on the graphite substrate. In this work, a 60 nm thick Ni film was formed to generate catalyst particles at 600 °C by the agglomeration mechanism. Then the CNTs were grown at 700 °C in the PECVD process using C_2_H_2_ as the carbon precursor. Their characterizations showed that the Ni catalysts particles were sparsely formed on the Ni film, and most of the catalyst particles have diameters of 100–170 nm. As a result, the large-sized catalyst particle results in the large CNT diameters around 100 nm. The sparse growth of CNT also degraded the vertical alignment during the synthesis. 

Zhou et al. [31] reported the synthesis of highly aligned vertical CNT array on a few layers of graphene on top of the SiO_2_ substrate. By reducing the Ni catalyst film to 1 to 2 nm, high-density CNT array was synthesized using the C_2_H_2_ precursor at 700 °C. An SEM image of the CNT array synthesized on the graphene/SiO_2_ substrate is shown in Figure 6b. One step forward, using the direct synthesis method, Jiang et al. [13] reported integration of the CNT via interconnect with the lateral graphene metal line in the double layer interconnect structure. The CNT via was grown on such few-layer graphene using C_2_H_2_ precursor in the PECVD process. The catalyst nanoparticles were formed with the 5 nm Ni film, which provided the CNT array with an average diameter of 40 nm. 

However, synthesis of the vertical CNT interconnects on 2D semiconductor materials are seldom reported. Ma et al. [32] reported the direct synthesis of CNT array on multilayer MoS_2_ films, which were transferred onto the SiO_2_ substrate using mechanical exfoliation. Using a multilayer Ni/Ti (3/1 nm) catalyst, multiwall CNTs were successfully grown on MoS_2_ at 550 °C. An SEM image of the CNT array synthesized on the MoS_2_/SiO_2_ substrate is shown in Figure 6c. In contrast, the 3 nm thick pure Ni catalyst film failed to facilitate CNT growth on MoS_2_ due to Ni diffusion into the MoS_2_ layer. Ti was found to form an alloy with MoS_2_ [57], as to serve as the diffusion barrier to facilitate the Ni catalyst nanoparticle formation. The density of CNT synthesized on MoS_2_ was estimated as 10^10^ cm^−2^, which was obtained by counting the number of de-wetted catalyst nanoparticles in a 1 μm^2^ area. Moreover, the intensity ratio I_D_/I_G_ <1 of the CNT synthesized on MoS_2_, shown in the Raman spectrum in Figure 6d, indicated normal lattice integrity of the CNT synthesized by the PECVD method. 

## 5. Summary

Table 1 summarized the reported high-density CNT array that synthesized using low temperature CMOS-compatible processes with process conditions listed for easy comparison. The reviewed experimental and theoretical works indicated that catalyst design and catalyst nanoparticle formation are critical in CNT growth. The thickness of the catalyst film assumes linear variation along with the average tube diameter and average wall number of the synthesized CNT array. A thin catalyst film turns out to be advantageous to synthesize high-density CNT array with small tube diameters. However, for the interconnect technology requiring a high wall-density, synthesis of multiwall CNTs with a large diameter can be preferred due to the small void ratio, which is caused by the hollow inner shell of the CNTs. The process of catalyst nanoparticle de-wetting process was found to be strongly affected by the composition of catalyst metal, the substrate, and the chamber environment. Particularly, diffusion of catalyst metal into the substrate unexpectedly degrades the nanoparticle density and morphology, which thus poses a challenge for in the growth of high-density and vertically aligned CNT array. Adding a few atomic layers of Al to the catalyst film and exposing to a trace of carbon improved the catalytic activity of the nanoparticles. Moreover, the alloyed catalyst nanoparticles generated using multilayer catalyst design showed additional advantages in reducing the process temperature by decreasing the activation energy of CNT growth. 

## Figures and Tables

**Table 1:** Characteristics of CNT array and synthesis conditions.

Ref	Catalyst/nm	Temp.	Precursor	Substrate	I_D_/I_G_	N_wall_ (cm^−2^)	N_tube_ (cm^−2^)
[14]	Ni-Al-Ni/ 1-0.5-1	340 °C	CH_4_	ZrO_2_	0.85	/	1 × 10^10^
	Ni-Al-Ni/ 1-0.5-1	450 °C	CH_4_	ZrO_2_	0.81	/	5 × 10^11^
	Ni/2	450 °C	CH_4_	ZrO_2_	/	/	1 × 10^11^
[27]	Co-Al-Mo/ 2.5-0.5-5	450 °C	C_2_H_2_	Cu	0.9	4.1 × 10^12^	1.3 × 10^11^
[29]	Co-Mo/ 2.5-0.8	450 °C	C_2_H_2_	Ti/Cu	1	7.8 × 10^12^	5.5 × 10^11^
	Co-Mo/ 2.5-0.3	450 °C	C_2_H_2_	Ti/Cu	0.9	4.8 × 10^12^	5.6 × 10^11^
	Co/2.5	450 °C	C_2_H_2_	Ti/Cu	0.8	2.4 × 10^12^	2.7 × 10^11^
[36]	Ni-Al-Ni/ 2-1-2	350 °C	CH_4_	TiSi	/	1 × 10^12^	2 × 10^10^
	Ni/4	350 °C	CH_4_	TiSi	/	/	5.9 × 10^9^
[38]	Co-TiN/ 0.5-0.5	450 °C	CH_4_	TaN	/	/	1 × 10^12^
[39]	Ni/0.6	400 °C	C_2_H_2_	TiN	/	1.2 × 10^13^	1.5 × 10^12^
[53]	Co-Al/3	350 °C	C_2_H_2_	Ti	0.83	/	5 × 10^10^
	Co/5	350 °C	C_2_H_2_	Ti	0.65	/	5 × 10^10^
